# Ferroptosis in diabetes mellitus and its complications: overview of clinical and preclinical research

**DOI:** 10.1038/s41420-025-02780-7

**Published:** 2025-11-06

**Authors:** Xiaoya Li, Meirong Fang, Xingyu Liu, Jingyi Jiang, Shengchen Wang, Xiaoshuang Mao, Zhongmei Zou, Wen Jin

**Affiliations:** 1 The Institute of Medicinal Plant Development, Chinese Academy of Medical Sciences & Peking Union Medical College, Beijing, China; 2 State Key Laboratory for Quality Ensurance and Sustainable Use of Dao-di Herbs, Beijing, China; 3 Key Laboratory of Bioactive Substances and Resource Utilization of Chinese Herbal Medicine, Ministry of Education, Beijing, China; 4 Key Laboratory of Efficacy Evaluation of Chinese Medicine against Glycolipid Metabolism Disorder Disease, State Administration of Traditional Chinese Medicine, Beijing, China

**Keywords:** Diabetes complications, Type 2 diabetes

## Abstract

Diabetes mellitus, a metabolic disorder of rising global incidence, imposes substantial health burdens through its systemic complications. Although the treatment strategies based on pathological changes and molecular mechanisms are constantly upgrading, the therapeutic effects, especially for complications, are not satisfactory. Emerging evidence highlights ferroptosis—an iron-dependent cell death pathway—as a critical regulator in diabetic pathophysiology. This review synthesizes clinical data, genetic studies, and therapeutic interventions across experimental models to establish ferroptosis’s multifaceted involvement in diabetes progression. Multiorgan analyses (pancreatic islets, heart, kidney, liver, brain, etc.) reveal ferroptosis-mediated pathways connecting localized tissue damage to systemic diabetic pathogenesis. Particularly, ferroptosis intersects with characteristic diabetic mechanisms, including oxidative stress, lipid peroxidation, and mitochondrial dysfunction. Our integrated assessment positions ferroptosis as a converging pathological mechanism in diabetes, proposing its molecular mediators as promising targets for innovative combination therapies. This mechanistic understanding could enable novel approaches for mitigating both metabolic dysregulation and end-organ damage in diabetes management.

## Facts

- Iron overload in the body increases the risk of developing diabetes.
- Ferroptosis contributes to the progression of diabetes and its complications.
- Inhibiting ferroptosis can improve diabetes and its complications.

## Open questions

- What evidence exists for the involvement of ferroptosis in patients with diabetes and its complications?
- How does ferroptosis mediate the onset of diabetes and its complications?
- What treatments are available to improve diabetes and its complications through the inhibition of ferroptosis?

## Introduction

Diabetes mellitus (DM) is a worldwide chronic metabolic disorder characterized by high blood sugar and glucose in the urine due to inadequate insulin production or reduced insulin sensitivity in target cells [1]. The prevalence of DM has increased due to the aging global population and changing lifestyles, currently affecting 589 million adults (20–79 years) [2]. The development of DM is associated with neuroendocrine disorders, insulin resistance, oxidative stress, chronic inflammation, and disruptions of gut microbiota. However, the precise mechanisms of DM remain to be further elucidated. In clinical practice, lifestyle modifications and hypoglycemic drugs are commonly used to manage DM. While these approaches have improved the disease over time, they have limitations such as the lack of a complete cure, the need for long-term medication, drug tolerance, and adverse effects. Therefore, there is a great need to delve deeper into the pathogenesis of DM and explore new therapeutic methods.

Ferroptosis, initially described by Dixon et al. is a form of nonapoptotic cell death characterized by its dependence on iron and induction by lipid reactive oxygen species (ROS) in 2012 [3]. The study demonstrated that inhibiting cystine uptake via the cystine/glutamate antiporter (system *x*_c_^−^) decreases glutathione (GSH) synthesis, impacting glutathione peroxidase 4 (GPX4) activity. This disruption causes a redox imbalance, leading to membrane lipid peroxidation, reactive oxygen species (ROS) accumulation, and ultimately, iron-dependent oxidative cell death [3]. Ferroptosis is distinct from programmed cell death processes such as pyroptosis, autophagy, and apoptosis, as it presents unique morphological and biochemical features. These cells exhibit smaller mitochondria, denser mitochondrial membranes, reduced or absent cristae, and ruptured outer mitochondrial membranes. Biochemically, cells are characterized by the buildup of iron ions, lipid peroxides, and lipid ROS deposition [4, 5].

Despite the growing interest in ferroptosis-DM crosstalk [6–15], current reviews lack a comprehensive analysis bridging clinical data, genetic studies, organ-specific mechanisms, and therapeutic translation. This review addresses three critical gaps: (1) By systematically integrating the research results of clinical data, genetic studies, and experimental models provides a relatively systematic and complete chain of evidence to prove the impact of ferroptosis on DM and its complications; (2) By systematically evaluating ferroptosis across 10 diabetic complications (e.g., cardiomyopathy, nephropathy, cognitive decline), we reveal shared *vs*. unique pathological motifs; (3) This work also involves the role of ferroptosis-targeting drugs in improving DM and related organs, and critically appraising ferroptosis-targeting drugs for their clinical feasibility beyond glucose control. Our synthesis establishes ferroptosis as a central hub connecting iron dyshomeostasis and multi-organ damage in DM, offering a framework for mechanistically informed therapies.

## The mechanism of ferroptosis

### Overview of ferroptosis

Among the various forms of cell death, apoptosis was originally defined as a programmed cell death mechanism on the basis of morphology, and the morphological hallmarks of apoptosis in tissue include cell shrinkage, nuclear fragmentation, and chromatin condensation [16]. The Stockwell group identified a novel small molecule, erastin, through a high-throughput screen for synthetic lethal compounds in 2003, which induced a nonapoptotic form of cell death [17]. Cells exposed to erastin do not display apoptotic nuclear morphology but exhibit altered mitochondrial structure, including loss of integrity, with other typical apoptotic features also missing [17]. Like erastin, a compound termed RSL3 can also induce nonapoptotic cell death [18, 19]. Further research has indicated that various iron chelators and lipophilic radical-scavenging antioxidants can inhibit this form of cell death, suggesting the role of the Fenton reaction and reactive oxygen species (ROS) [18, 19]. In 2012, Dixon et al. coined the term ‘ferroptosis’ to describe this mode of cell death, which is characterized by its iron dependency and unique morphology, biochemistry, and genetics, distinguishing it from apoptosis and other forms of cell death [3]. In 2018, the Nomenclature Committee on Cell Death characterized ferroptosis as a type of regulated cell death caused by oxidative disruption within the intracellular microenvironment that is consistently regulated by GPX4 [20]. Recent developments have greatly improved our understanding of the mechanisms underlying ferroptosis and its implications in a range of diseases.

### Occurrence mechanism of ferroptosis

**Fig. 1:**
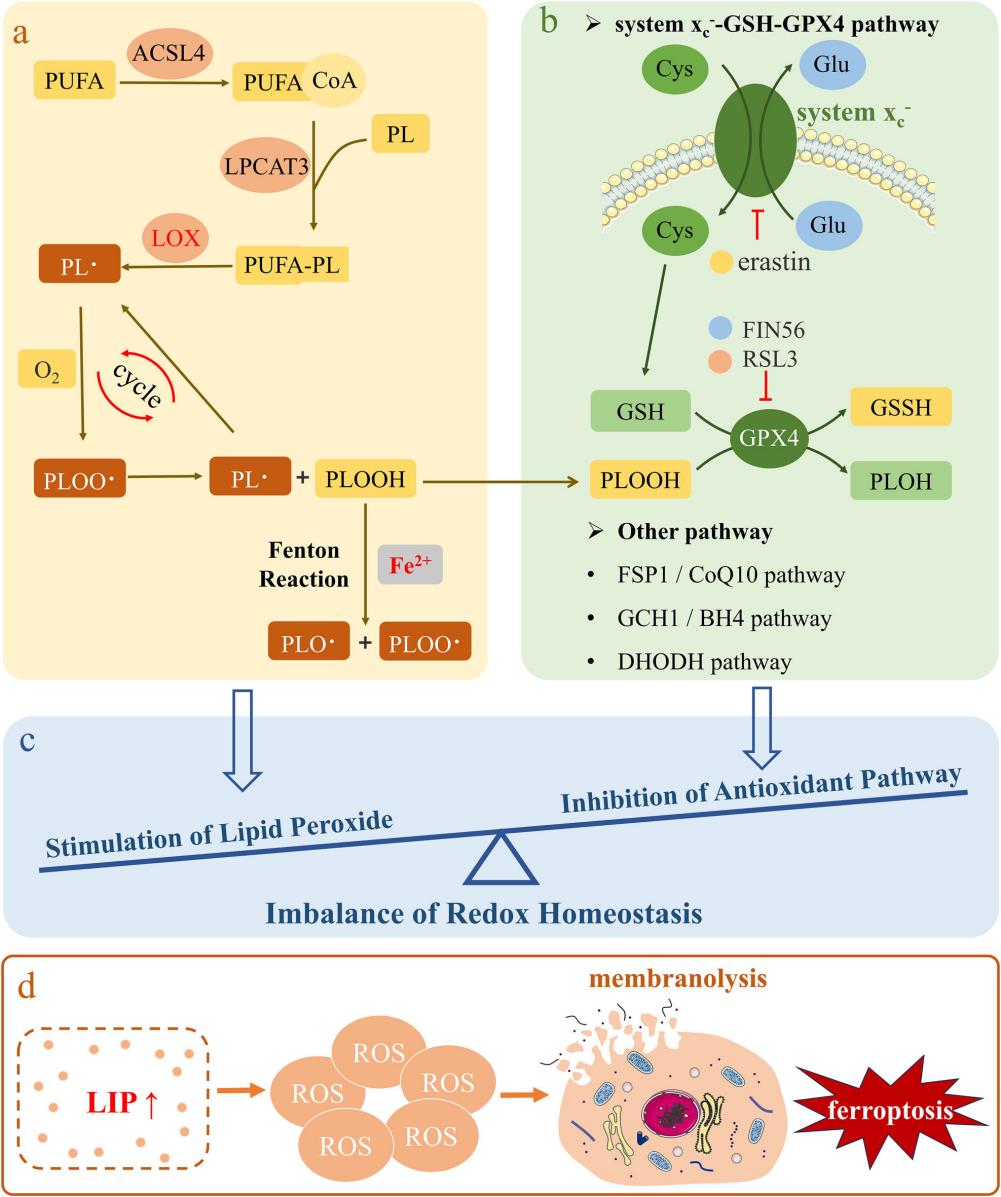
Molecular mechanism of ferroptosis. **a** In this process, polyunsaturated fatty acids are integrated into the cell membrane by reacting with phospholipids, which is catalyzed by ACSL4 and LPCAT3. This is followed by the generation of lipid ROS under the influence of the LOX enzyme, leading to the propagation of lipid peroxides and lipid ROS. Ferrous ions further exacerbate this situation by reacting with lipid peroxides, resulting in increased lipid ROS. **b** Conversely, cells employ a classical detoxification pathway to counteract lipid peroxide accumulation, primarily through a GPX4-dependent antioxidant mechanism. In this pathway, lipid peroxides are converted to nontoxic lipid alcohols with the help of glutathione and GPX4 enzymes. However, when GPX4 is inhibited by small molecules such as FIN56 and RSL3 or when the glutathione transporter is blocked by erastin, antioxidant defense is compromised. **c** The essence of ferroptosis is an imbalance in intracellular redox homeostasis, which occurs when cell membrane lipids undergo peroxidation due to the direct or indirect influence of iron, coupled with the inactivation of one or more antioxidant pathways. **d** As a result, membrane lipid peroxides and ROS accumulate, ultimately causing rupture of the cell membrane and leading to cell death.

Ferroptosis is a result of cellular metabolism and an imbalance in redox homeostasis (Fig. 1). The peroxidation of cell membrane lipids, triggered by iron and the inactivation of antioxidant pathways, results in the accumulation of lipid peroxides and reactive oxygen species (ROS), causing cell membrane rupture and cell death. Ferroptosis, driven by iron-dependent lethal lipid peroxidation, can be inhibited through direct blockade of lipid peroxidation or iron depletion via pharmacological or genetic approaches [21].

#### Iron and lipid peroxidation

##### Lipid peroxidation

Ferroptosis is a distinct form of oxidative stress marked by the accumulation of lethal lipid peroxides in membranes, particularly phospholipid hydroperoxides (PLOOHs), a type of lipid-based reactive oxygen species [22]. Phospholipids, together with sphingolipids and cholesterol, constitute the primary components of the cell membrane. The diversity of phospholipids (PLs) arises from the various combinations of two fatty acyl chains and the head group, with one chain potentially containing polyunsaturated fatty acids (PUFAs) [23]. PUFAs are susceptible to peroxidation because of the weak C‒H bonds in the central methylene group of their bisallylic structure (–CH=CH–CH2–CH=CH–). Therefore, PUFA moieties always serve as necessary substrates for the peroxidation of ferroptosis when they are incorporated into lipids on cell membranes. Acyl-CoA synthetase long-chain family member 4 (ACSL4) and lysophosphatidylcholine acyltransferase 3 (LPCAT3) are involved in this process [24]. Previous studies have shown that the ACSL enzyme family is essential for ferroptosis, highlighting that fatty acid activation to CoA ester is a key regulatory step in ferroptosis [25–27]. The activation of ACSL4 facilitates the conversion of PUFAs to PUFA-CoA, which LPCAT3 then esterifies into PLs, leading to the formation of membrane PLs with PUFAs.

The buildup of oxidized lipids containing polyunsaturated fatty acids (PUFAs) in cell membranes triggers ferroptosis (Fig. 1) [22]. The peroxidation of phospholipids (PLs) involves three stages: initiation, propagation, and termination [23]. Initially, lipoxygenases (LOXs) extract a hydrogen atom from the diallyl position of a PUFA, resulting in the formation of a phospholipid radical (PL•). During the propagative phase, molecular oxygen reacts with phospholipid radicals rapidly, leading to the formation of phospholipid peroxy radicals (PLOO•). The PLOO• abstracts a hydrogen atom from an adjacent PUFA, forming PLOOH and a new PL•. The newly synthesized PL• can then undergo the propagation step again, generating more PLOOHs and PL•. Eventually, the radical chain and propagative phases cannot continue until termination occurs due to the lack of available lipid substrates or the reduction of oxidized lipids by cellular antioxidants [23].

##### The role of iron

Lipid peroxidation is facilitated by the labile iron pool, which enhances the Fenton reaction to propagate lipid peroxidation, and by iron-dependent enzymes that initiate lipid hydroperoxide formation [22]. In cells, iron is typically bound in complexes such as heme, iron‒sulfur clusters (enzyme cofactors), and ferritin, with only a small portion existing as the labile iron pool (LIP) [23]. Labile iron reacts with hydrogen peroxide (H_2_O_2_) in the Fenton reaction, producing toxic hydroxyl radicals. Like H_2_O_2_, PLOOH can also generate toxic radicals (PLO• and PLOO•) through an iron-catalyzed Fenton reaction. These radicals can engage in lipid peroxidation chain reactions, resulting in the accumulation of ROS [28]. Cellular processes that increase the labile iron pool, including autophagic degradation of ferritin, transferrin uptake, and ferroportin inhibition, can sensitize cells to ferroptosis [29–31]. Iron-dependent enzymes, including lipoxygenases (LOXs) and cytochrome P450 oxidoreductase (POR), are involved in promoting lipid peroxidation. LOXs are nonheme iron-dependent enzymes implicated in PUFA peroxidation and ferroptosis, with arachidonate 15-lipoxygenase (ALOX15) playing a significant role [32–35]. Recent findings also indicate that POR, a heme-containing enzyme family, contributes to lipid peroxidation during ferroptosis by initiating this process [36].

#### Antioxidative metabolic pathways

##### GPX4-dependent antioxidative pathway (system *x*_c_^−^–GSH–GPX4)

The anti-lipid peroxidation pathway mediated by GPX4 is the classical pathway for effectively neutralizing the accumulation of PLOOH and lipid ROS in cells (Fig. 1). GSH, a thiol-containing tripeptide (γ-L-glutamyl-L-cysteinylglycine), is a crucial cellular reducing agent and serves as a cofactor for enzymes such as GSH-dependent glutathione transferases (GSTs) and glutathione peroxidases (GPXs). GPX4 catalyzes the conversion of toxic PLOOH to nontoxic PLOH, counteracting lethal lipid peroxidation. This process involves the catalytic selenocysteine residue of GPX4 and typically utilizes two electrons supplied by GSH [37]. Cysteine, which is essential for glutathione synthesis, is acquired primarily in its oxidized form, cystine, via the cystine/glutamate antiporter (system *x*_c_^−^, comprising the subunits SLC7A11 and SLC3A2), or it is synthesized from methionine and glucose through the trans-sulfidation pathway. Ferroptosis may be induced when system *x*_c_^−^-mediated Cys2 import is inhibited by physiological conditions, such as elevated extracellular glutamate, or by small molecules such as erastin [3, 38]. Ferroptosis can be induced by genetically deleting GPX4 or by using small molecules that either covalently inhibit GPX4 (e.g., RSL3) or promote its degradation (e.g., FIN56) [33, 39, 40]. Ferroptosis typically involves GSH depletion or GPX4 inactivation, alongside the accumulation of PLOOH and lipid ROS.

##### GPX4-independent antioxidative pathways

While the system *x*_c_^−^–GSH–GPX4 axis is acknowledged as the main antilipid peroxidation pathway in ferroptosis, recent studies have identified three GPX4-independent mechanisms that also inhibit ferroptosis: ferroptosis suppressor protein 1 (FSP1)/CoQ10, GTP cyclohydrolase 1 (GCH1)/tetrahydrobiopterin (BH4), and dihydroorotate dehydrogenase (DHODH). The FSP1/CoQ10 pathway suppresses lipid peroxidation and ferroptosis by utilizing FSP1 to regenerate reduced CoQ10, a lipophilic radical-trapping agent, via NADPH. This process inhibits lipid ROS spread at the plasma membrane and indirectly regenerates oxidized α-tocopheryl radical (vitamin E), a potent antioxidant, to reduce lipid ROS [41, 42]. A previous study indicated that GCH1 prevents ferroptosis via its metabolites, tetrahydrobiopterin (BH4) and dihydrobiopterin (BH2). BH4 functions as a direct radical-trapping antioxidant and is involved in ubiquinone synthesis [43, 44]. DHODH acts as a mitochondrial suppressor of ferroptosis by reducing mitochondrial CoQ10, similar to the role of FSP1 in membranes [45, 46]. Inactivation of one or more of these mechanisms causes unchecked lipid peroxidation, compromising membrane integrity and triggering ferroptosis.

## Ferroptosis in diabetes mellitus

### Ferroptosis in diabetes clinical studies

#### Iron overload is correlated with increased diabetes risk by multi-level evidence

DM is a chronic condition characterized by persistently high blood glucose levels beyond the normal range. DM can be categorized into type 1 (T1DM) and type 2 (T2DM) on the basis of insufficient insulin secretion and insulin resistance. Regardless of the type, elevated blood sugar levels can lead to damage in various systems and organs of the body, resulting in complications such as cardiovascular diseases, neuropathy, kidney diseases, retinopathy, and foot complications. Based on the current understanding, oxidative stress is a key pathological process of diabetic complications, resulting in lipid peroxidation and mitochondrial dysfunction. However, the therapeutic effect of DM, especially on its complications, is not satisfactory.

Recent research has revealed a potential link between iron metabolism and DM development. This assumption originated from the observation that individuals with hemochromatosis, a genetic condition marked by excessive iron accumulation, exhibit a notably higher incidence of T2DM [6, 47]. Then, strong evidences from Mendelian randomization study [48], systematic review and meta-analysis [6, 9], cohort studies [7], prospective nested case-control study [8], observational studies [11] etc., indicated that elevated iron storage, ferritin, transferrin saturation, iron stores and decreased transferrin levels, is linked to a heightened risk of DM and its complication. Consistent with the results of the risk analysis, a cross-sectional study [10] and a longitudinal, retrospective study [14] also found that elevated serum ferritin and reduced serum transferrin were associated with DM and diabetic end-stage renal disease. The retrospective study also found consistency between ferritin and previous results. However, the correlations of transferrin and transferrin saturation with T2DM showed an inverse trend [15]. This inconsistency might be due to the fact that retrospective studies are vulnerable to factors such as incomplete control of confounding factors, recall bias, and data subjectivity. Furthermore, stratification analyses and other studies have also concluded that the correlation between iron metabolism and DM is gender-related [11, 13]. In addition, a follow-up study by Beata et al. found that, independent of other variables, serum ferritin and transferrin saturation can strongly predict 5-year all-cause mortality rates in DM patients [12], providing strong evidence for the association between ferroptosis and DM. In summary, based on multi-level evidence, we found that iron metabolism imbalance induced by iron overload is a risk factor for DM, and that addressing the pathological process caused by iron overload may improve disease management.

#### Iron overload-mediated β-cell dysfunction, insulin resistance and oxidative stress

While iron overload is associated with an increased risk of T2DM, the precise mechanisms by which iron metabolism affects DM potentially involve pancreatic β-cell damage, insulin resistance, and oxidative stress.

The pancreatic islet of β-cells is susceptible to ferroptosis. Owing to the low expression of the H_2_O_2_-detoxifying enzyme catalase and highly abundant GPX4, in contrast with its low expression in other islet cell types, β-cells may theoretically be susceptible to ferroptosis. Furthermore, Research indicates that iron accumulation occurs in both acinar cells and pancreatic islet β-cells in iron-loaded human pancreatin [49–51]. Therefore, by damaging the β-cells [49], ferroptosis directly affects insulin production, thereby promoting the occurrence of DM at the root cause.

Iron overload exacerbates the development of insulin resistance and oxidative stress in diabetic patients. Current studies on T2DM suggest that insulin resistance in adipose tissue, liver, and muscle is affected by ferroptosis [52]. Excess hepatic iron storage contributes to liver steatosis, elevates plasma transaminase activity, and triggers oxidative stress [53]. Iron imbalance in adipose tissue influences glucose metabolism by affecting adipocyte differentiation, tissue growth, adipokine secretion, lipid synthesis, and lipolysis [54]. A study reported that the serum ferritin level may also serve as an early indicator of adipose tissue dysfunction, as it is associated with elevated lipid metabolism markers [55]. Adiponectin plays a role in regulating insulin sensitivity. Research indicates that elevated serum ferritin levels in T2DM patients are associated with reduced adiponectin levels, suggesting a potential link between iron overload and insulin resistance in adipose tissue [56]. Additionally, heme levels are closely related to iron overload. Moreno et al. reported that heme levels and export were significantly increased in adipose tissue in T2DM patients and that the gene level of heme exporter (FLVCR1) was positively correlated with fasting blood glucose, suggesting that systemic glucose impairment during T2DM may be associated with heme export from adipose tissue [57]. Research has shown that heightened oxidative stress in individuals with DM is linked to iron imbalances due to decreased iron-binding antioxidant capacity [58]. Numerous studies have demonstrated that in T2DM patients, plasma GPX4 levels are reduced, whereas ACSL4, MDH, 4-HNE, and ROS levels are significantly elevated, indicating a strong link between oxidative stress in DM and ferroptosis [59–62]. The same findings were also reported in the kidney, testis, and other tissues of diabetic patients [63, 64]. A prospective observational study indicated that combining GPX4, ACSL4, MDA, and ROS could effectively predict diabetic nephropathy [65]. In addition, nonenzymatic glycosylation of transferrin and deamidation of ferro oxidase protein (CP) induced by high glucose decrease the stability of transferrin, disrupt Fe^3+^/Fe^2+^ homeostasis, and aggravate oxidative stress in T2DM patients [66]. Research indicates that elevated fibromodulin expression in diabetic patients may increase the level of intracellular iron and oxidative stress by promoting the internalization and degradation of iron transporters in intestinal cells and monocytes [67]. In addition, Tatsch et al. reported that iron homeostasis in mitochondria caused by frataxin deficiency can aggravate oxidative stress in T2DM patients [68].

#### Diabetic patients expressed differential genes related to ferroptosis metabolism

Numerous studies have demonstrated strong associations between the ferroptosis signaling pathway, its related genes, and the onset of DM, with potential implications even for offspring [69–71]. Gene sequencing technology has revealed that the hub genes HIF1A, HILPDA, and SCD are closely associated with ferroptosis, T2DM, hypoxia, and lipid metabolism [69]. Significant differences in ferroptosis-related genes were observed between T2DM patients and non-T2DM patients, and five key genes (JUN, NFE2L2, ATG5, KRAS, and HSPA5) involved in the pathogenesis of T2DM-related β-cell dysfunction were identified [72]. In addition, Ye et al.’s bioinformatics analysis identified microsomal glutathione S-transferase 1 (MGST1) as a potential key gene involved in ferroptosis related to islet dysfunction in T2DM [71]. Research has revealed a potential link between mutations in ferroprotein Q248H, heme oxygenase (HO) activity, and an elevated risk of T2DM. Epidemiological studies suggest that offspring of T2DM patients may have an elevated metabolic risk for oxidative stress and iron-mediated cardiovascular disease, potentially due to the inheritance of specific genes [73–76]. Moreover, the TMPRSS6 gene is related to iron metabolism and plays a role in regulating iron absorption in the intestine and iron storage and release in the liver. Certain TMPRSS6 SNPs are associated with an increased risk of gestational DM in Chinese Han pregnant women [77], while they correlate with a reduced risk of T2DM in American men but show no association in American women [78]. Although both studies explored the association of TMPRSS6 polymorphisms with DM, finding that rs855791 and rs4820268 were linked to iron metabolism markers and the former showed sex-specific risk differences—possibly due to metabolic, hormonal, and physiological disparities between sexes—they had limitations including uncontrolled ethnic variations, sample constraints, and uninvestigated mechanisms beyond iron metabolism. In a subsequent study, Zhang et al. further demonstrated that GLUT1 exacerbates trophoblast ferroptosis by regulating AMPK/ACC-mediated lipid metabolism, thereby promoting fetal growth restriction associated with gestational DM [79].

Furthermore, variations in the expression of ferroptosis-related genes were observed in certain patients with diabetic complications. For example, urine metabolomics analysis revealed that metabolites associated with the ferroptosis signaling pathway were uniquely expressed in patients with diabetic nephropathy, indicating that ferroptosis could be a key mechanism in disease progression [80]. Another study revealed that the ARNTL gene is overexpressed in diabetic kidney disease patients [81], whereas the HINT2 gene is downregulated [82]. Animal experiments have verified and studied the specific mechanisms involved, suggesting that these two genes may be potential targets for treating diabetic kidney disease [81, 82]. In addition, Pan et al. suggested that targeting ferroptosis is essential for understanding and treating periodontitis in the context of T2DM. Key genes associated with ferroptosis, namely, IL-1β, IL-6, NFE2L2, and ALOX5, facilitated the identification of 13 potential therapeutic drugs for diabetic periodontitis, including echinacea and ibuterast [83].

In summary, a large amount of clinical evidence shows that iron storage indicators, oxidative stress-related indicators, and ferroptosis-related genes are related to the occurrence of DM and can be used as targets for diagnosis and treatment. Although many epidemiological and genetic studies have been conducted in clinical practice, the specific molecular mechanism by which ferroptosis mediates the increased risk of DM has not been extensively studied in humans, and more studies have been carried out in animal and cell models.

### Ferroptosis in diabetes mellitus models

We examined ferroptosis in animal and cellular models to explore its role in DM and related complications. Commonly used animal models of DM include STZ-induced diabetic mouse models and spontaneous db/db diabetic mouse models. The tissues associated with DM and its complications include the islets, liver, kidney, heart, brain tissue, etc. The observation and study of ferroptosis in diabetic cell models have focused mainly on the cells corresponding to the above tissues. This section reviews the occurrence and development of ferroptosis in DM-related tissues and complications, integrating both cell and animal models owing to their frequent interconnection. The improvement of DM and its complications by inhibiting ferroptosis in diabetic tissues in drug research is summarized in the table (Table 1).

**Table 1:** Drugs for ameliorating diabetes by inhibiting ferroptosis.

Drugs	Experimental models	Affected tissues	Related pathways	Functions	Ref.
Metformin	HG/PA-induced NIT-1, HG/PA-induced mouse islets	Islet	\	GPX4↑ ACSL4↓	[84]
1,25-dihydroxyvitamin D3	HG-induced INS-1, HFD/STZ-induced T2DM rat	Islet	FOXO1	GPX4↑ FOXO1, Iron, ROS, ACSL4↓	[86]
MSC-EXO	HFD/STZ-induced T2DM mouse	Islet	AKT/ERK/Nrf2	AKT, ERK, NRF2, GPX4, SLC7A11↑ MDA↓	[90]
Eugenol	STZ-induced DM mouse	Islet	Nrf2/HO-1	NRF2, HO1, GPX4, SLC7A11↑Fe^2+^↓	[91]
1,8-Cineole	HGHF/STZ-induced T2DM mouse, HG-induced mouse islet β cells	Islet	PI3K/AKT/mTOR	PI3K, p-AKT, p-mTOR, GPX4, COX2↑ Fe^2+^, lipid ROS↓	[92]
6-gingerol	HG/HF-induced H9c2, STZ-induced DM mouse	Heart	Nrf2/HO-1	Nrf2, HO-1, GPX4↑ Iron, FACL4, IL -1 β, IL-6, TNF-α↓	[95]
Sulforaphane	HFD/STZ-induced T2DM mouse	Heart	Nrf2	NRF2, GPX4, FTH1, FPN1↑ Fe^2+^↓	[98]
NaHS	HG/PA-induced HL-1, db/db	Heart	Nrf2/GPX4/GSH	Promote Syvn1–Keap1 interaction GPX4, GSS, SLC7A11↑ Fe^2+^, MDA, TFR1↓	[110]
Isorhapontigenin	db/db	Heart	PRDX2/MFN2/ACSL4	Inhibit mitochondria-associated ferroptosis	[111]
Baicalin	db/db, HG-induced H9c2	Heart	SENP1/SIRT3	SENP1, GSH, SLC7A11, GPX4↑ Iron, MDA, PTGS2↓	[112]
Dietary capsaicin	HFD/STZ-induced T2DM mouse, HG/hypoxia-induced AC16 cardiomyocytes	Heart	TRPV1 and Nrf2/HMOX1	TRPV1, NRF2, HMOX1 GPX4, GSH↑ LDH, MDA↓	[113]
Retinoic acid	STZ/HFD-induced DM rat, HG-induced HUVEC	Blood vessel	PI3K/AKT/YAP	YAP, GPX4↑ PI3K, AKT, ROS, Fe^2+^↓	[114]
Schisandrol B	HFD/STZ-induced DM mouse, HG-induced H9c2	Heart	p53	SLC7A11, GPX4, GSH↑ p53, Fe^2+^, MDA, ROS↓	[115]
Rosiglitazone	STZ-induced DM mouse	Kidney	ACSL4	ACSL4, Iron, MDA, ROS, IL-6, TNF-α↓ GPX4↑	[116]
Umbelliferone	db/db, HG-induced HK-2	Kidney	Nrf2/HO-1	Nrf2/HO-1, GPX4, GSH↑ Iron, MDA, ROS↓	[119]
Fenofibrate	STZ-induced DM mouse, HG-induced HK-2	Kidney	Nrf2	NRF2, GPX4, FTH1, SLC7A11↑ Iron, MDA, TFR1↓,	[120]
Paricalcitol	db/db, HG-induced PTECs	Kidney	VDR/Nrf2/HO-1	Nrf2, HO-1, GPX4, GSH, SLC7A11↑ Iron, MDA, ROS, TFR1, FTH1↓	[121]
Carnosine	STZ-induced DM mouse, HG-induced HK-2	Kidney	Nrf2	NRF2, GPX4, GSH↑ Fe^2+^, ACSL4, MDA, ROS↓	[122]
Leonurine	STZ/HFD-induced DM mouse, HG-induced HUVEC	Kidney	Nrf2	NRF2, GPX4, GSH, FTH1, FTL↑ Fe^2+^, MDA↓	[123]
Dapagliflozin	db/db, HG/HF-induced HK-2	Kidney	HIF1-α/HO1	HIF1-α/HO1, Iron, MDA, TFR1↓ GPX4↑	[124]
Triptolide	db/db, HG-induced HK-2	Kidney	Nrf2	NRF2, GPX4, GSH, FTH1, SLC7A11↑ Fe^2+^, MDA, TFR-1↓	[124]
DDO-1039	STZ/HFD-induced DM mouse, db/db, HGHF-treated human podocytes	Kidney	Nrf2/HO-1	NRF2, HO-1, NQO1, GPX4↑	[125]
Potentilla Discolor Bunge	STZ-induced DM rat, HGHF-induced HK-2	Kidney	Nrf2/HO-1	NRF2, HO-1, GPX4, GSH, SLC7A11↑ Fe^2+^, MDA, ROS↓	[126]
Swietenine	STZ/HFD-induced DM mouse, HG-induced MPC5	Kidney	Akt/GSK-3β/Nrf2	AKT, GSK-3β, NRF2, GPX4↑ Fe^2+^, ACSL4, MDA, ROS↓	[127]
Canagliflozin	db/db, HG-induced HK-2	Kidney	FOXA1-CPT1A	FOXA1/CPT1A↑ GPX4, SLC7A11↑	[131]
Shen Kang Pill	HFD/STZ-induced T2DM mouse	Kidney	HIF1-α/HO-1	HIF1-α/HO1↓ Fe^2+^, MDA, ROS↓, GPX4↑	[132]
Rhein	STZ-induced DM mouse, HG-induced podocyte	Kidney	Rac1/NOX1/β-catenin	Rac1/NOX1/β-catenin↓ Fe^2+^, ROS, MDA, TFR1, ACSL4↓	[134]
Dapagliflozin	STZ-induced DM mouse, HG/PA-induced HK-2	Kidney	SLC40A1	GPX4, GSH, SLC7A11↑ Iron, MDA↓	[139]
Calycosin	db/db, HG-induced HK-2	Kidney	\	GPX4, GSH↑ ROS, MDA, LDH, NCOA4↓	[141]
Dulaglutide	db/db	Kidney	\	GPX4↑ ACSL4, SLC7A11, Ptgs2, 4-HNE↓	[142]
Cordycepin	STZ-induced DM mouse, HG-induced MPC5	Kidney	\	SLC7A11, GPX4↑	[143]
Kidney tea (*Orthosiphon aristatus* (Blume) Miq.)	db/db	Kidney	\	Improved mitochondrial damage MDA, 4-HNE, ACSL4, NCOA4↓ SOD, GPX4, FTH1↑	[144]
Curcumin nanocrystals	STZ-induced DM rat, HG-induced HK-2	Kidney	\	GSH, GPX4, SLC7A11, FTH-1↑ LDH, MDA, Iron, ROS, NCOA4, TFR-1↓	[145]
Vitamin D	HFD-induced DM mouse, HG-induced HK-2	Kidney	Klotho/p53	Klotho, SLC7A11, GPX4↑, p53, TFR1, ACSL4↓	[147]
Liraglutide	db/db, HG-induced HK-2	Kidney	Fsp1/CoQ10/NAD(P)H	FSP1, GPX4, FPN1, SOD, GSH↑TFR1, MDA, LPO, 8-OHDG, 4-HNE, 12-LOX, NOX4↓	[148]
Hirsutine	db/db	Kidney	p53/GPX4	p53, GPX4↑ Iron, ROS, MDA↓	[149]
Qing-Re-Xiao-Zheng-(Yi-Qi) formula	STZ-induced DM mouse, HG-induced MPC5	Kidney	AMPK/ACC1	AMPK, GPX4, GSH, SLC7A11, SOD↑ ACC1, Fe^2+^, ACSL4, MDA, ROS↓	[150]
Liraglutide	db/db, HG-induced HepG2	Liver	Nrf2/HO-1/GPX4	Nrf2/HO-1/GPX4, SLC7A11, SOD, GSH-PX, GSH↑ Iron, MDA, 4-HNE, NOX4, ROS↓	[153]
Liraglutide	db/db	Brain	SLC7A11/GPX4	ROS, MDA, TRF1, ACSL4↓ GPX4, SLC7A11, FTH, FPN1↑	[156]
Gemfibrozil	db/db, HG-induced astrocyte	Brain	PPARα/SLC7A11/GPX4	Iron, ROS, 4-HNE, TRF1, GSSG↓ FPN, FTL, GSH, SLC7A11, GPX4↑	[160]
Artemisinin	HFD/STZ-induced T2DM mouse	Brain	Nrf2	Nrf2, HO-1, GPX4↑ ROS, MDA, GSH, Fe^2+^↓	[162]
Dendrobine	db/db, AGEs-induced HT22	Brain	Nrf2/HO-1/GPX4	Improves mitochondrial dynamics, Iron, MDA, TRF1↓ Nrf2/HO-1/NQO1, GPX4, FTH, FPN1↑	[163]
TAC	HGHF/STZ-induced T2DM rat	Brain	NLRP3	NLRP3↓ GPX4, SLC7A11↑	[164]
Salidroside	db/db	Brain	PPARγ	PPARγ, NRF2, GPX4↑ ROS, MDA, Fe^2+^↓	[165]
Resveratrol	HFD/STZ-induced T2DM mouse	Brain	miR-9-3p/SLC7A11	miR-9-3p, iron↓ GPX4, SLC7A11, FTH, SOD, GSH↑	[166]
Corilagin	STZ-induced T2DM mouse, HG-treated ARPE-19	Retina	Nrf2	NRF2, HO-1, GPX4, SOD, GSH, MPO↑ Fe^2+^, MDA, ROS↓	[170]
Resveratrol	STZ-induced T2DM mouse, HG-treated HRCECs	Retina	SIRT1/HMGB1	SIRT1, GPX4, GSH, SLC7A11↑ HMGB1, Fe^2+^, MDA↓	[171]
Carnosic acid	STZ-induced DM rat, HG-induced HRMEC	Retina	SIRT1/p53/SLC7A11	SIRT1, p53, GPX4, SLC7A11↑ Fe^2+^, Iron↓	[172]
Fenofibrate	STZ-induced DM RAT, HG-treated ARPE-19	Retina	P53/FSP	P53↓, FSP1↑	[174]
Poliumoside	HGHF/STZ-induced T2DM mouse, HGHF-induced BMSC	Bone	Nrf2/HO-1/GPX4	Nrf2, HO-1, GPX4, GSH↑ MDA, ROS, ACSL4↓	[176]
Irisin	STZ-induced T1DM mouse, HG-induced H9c2	Heart	SIRT1-p53-SLC7A11/GPX4	SIRT1, SLC7A11, GPX4↑ p53, MDA, LPO, ROS↓	[177]
Irisin	STZ-induced T1DM mouse, HG-induced MC3T3-E1	Bone	eIF2α/ATF4/C/EBP- CHOP	FNDC5, GPX4, SLC7A11, FTH↑ p-elF2α, ATF4, CHOP↓	[177]
Guhan Yangsheng Jing	STZ-induced DM rat	Teste	Nrf2/HO-1	NRF2, HO-1, GPX4, SLC7A11, GSH, SOD↑ Iron, MDA, ROS, LPO↓	[179]
Isorhamnetin	STZ-induced DM rat, HG-induced corpus cavernosum endothelial cells	Penis	HMOX1	GPX4, HMOX1, SOD↑	[180]
Icariin	STZ-induced T1DM rat, HG-induced HUVEC	Penis	\	GPX4, GSH, SOD↑ Iron, MDA, ROS, ACSL4↓	[181]
Hesperidin	STZ-induced DM rat, HG-induced HUVEC	Penis	Nrf2/HO-1/GPX4	Nrf2, HO-1, GPX4↑ ROS↓	[182]
Berberine	AGEs-induced HaCaT, db/db	Skin	Nrf2	Nrf2, GPX4, FTL, FTH↑ Fe^2+^, MDA, ROS↓	[184]
Orientin	STZ/HFD-induced DM mouse, HG-induced HUVEC	Blood vessel	Nrf2/GPX4	NRF2, GPX4, GSH↑ MDA, ROS↓	[185]
Dulaglutide	db/db, HG-induced HaCaT	Skin	Nrf2	NRF2, GPX4, SLC7A11, SOD, GSH↑ Fe^2+^, MDA, ROS↓	[186]
Dracorhodin	HGHF/STZ-induced DM rat, HG-induced HUVEC	Skin	Nrf2	Nrf2, HO-1, GPX4, SLC7A11, SOD, GSH↑ Fe^2+^, MDA, ROS↓	[187]
Salidroside	db/db	Heart	Gut microbiota	Probiotic bacteria↑, ↓, Iron↓	[189]
Astragaloside IV	db/db	Kidney/Colon	Entero-renal axis	*Akkermansia, Ligilactobacillus* and *Lactobacillus*↑	[190]

This table summarizes the research on various drugs that improve diabetes by inhibiting ferroptosis. The experimental models include cell models induced by HG/HF, as well as animal models induced by STZ/HFD, etc. It shows the tissues on which the drugs act, involving islets, heart, kidney, liver, brain, bone, etc.; the related ferroptosis pathways, such as GPX4/ACSL4, Nrf2/HO-1, etc.; and the therapeutic effects, represented by the up-regulation (“↑“) or down-regulation (“↓“) of related factors.

#### Ferroptosis in the pancreatic islets of diabetic models

Iron overload and ferroptosis significantly impact the function and survival of pancreatic β-cells in DM, and ferroptosis in β-cells may be more pronounced in patients with a longer disease course [84]. Previous research has indicated that excess iron causes oxidative stress in β-cells, leading to reduced insulin secretion due to β-cell apoptosis and desensitization to glucose-induced insulin secretion [85]. Specifically, ferroptosis occurs in the pancreatic tissue of T2DM model rats and high glucose-induced INS-1 cells, with increased iron content and expression of ROS and ACSL4, while GPX4 expression is downregulated [86]. Research has shown that drugs can improve iron-induced damage, thereby ameliorating pancreatic damage in DM models. For example, Sun et al. reported that metformin (Met) treatment alleviated ferroptosis in β-cells in T2DM patients by increasing GPX4 expression and downregulating ACSL4 expression, whereas RSL3 injection significantly diminished the antiferroptotic effect of Met [84]. Moreover, Stancic et al. administered the ferroptosis inhibitor ferrostatin 1 (Fer-1) to diabetic mice, which reduced islet monocyte infiltration, serum glucose levels, the incidence of hyperglycemia, and the production of 4-HNE, a lipid peroxide byproduct, suggesting that inhibition of ferroptosis positively affects β-cell protection and survival under diabetic conditions [87].

Research has identified potential molecular mechanisms of ferroptosis in β-cells within diabetic contexts. Exposure to elevated iron and glucose levels significantly decreased SNAP-25 protein expression in the MIN6 mouse pancreatic β-cell line [88]. Stancic et al. used the rat pancreatic beta cell line Rin-5F to induce cell death via DM mimics, including high glucose, H_2_O_2_, and STZ, and reported that these factors accumulate ROS and iron, inactivate NRF2, and reduce the mitochondrial membrane potential, while treatment with Fer-1 mitigated these effects [87]. Furthermore, in STZ-induced T2DM model rats, JUN protein expression was increased, and NRF2 expression was decreased in pancreatic tissues, potentially linking these changes to ferroptosis in T2DM-related β-cell dysfunction [72]. In addition, Sun et al. reported that Met mitigated HGHF-induced ferroptosis in NIT-1 cells by regulating the GPX4/ACSL4 axis, resulting in an antiferroptotic effect similar to that of RAS3 [89]. Overall, research on ferroptosis-related proteins in the pancreas associated with DM is quite limited. In addition to proteins such as SNAP-25, NRF2, and JUN, other proteins and pathways involved in the crosstalk with ferroptosis pathways have yet to be identified.

Some studies report that inhibiting ferroptosis can improve pancreatic islet functional injury in DM. Exosomes derived from mesenchymal stem cells(MSC-EXO) were found to regulate the AKT/ERK signaling pathway by delivering bioactive proteins, thereby inhibiting NRF2-mediated ferroptosis and improving the function and quantity of β cells, which may serve as a novel drug carrier for islet-targeted therapy [90]. Eugenol significantly alleviates the downregulation of GPX4 and antioxidant Nrf2 in diabetic-induced pancreatic tissues, protecting β-cell viability and function by inhibiting ferroptosis [91]. 1,8-Cineole exerts its anti-ferroptotic effect in diabetic β cells by activating the PI3K/AKT/mTOR pathway [92].

#### Ferroptosis in the heart of diabetic models

Diabetic cardiomyopathy, a frequent DM complication, involves myocardial structural and functional abnormalities resulting from prolonged poor blood sugar control, potentially leading to heart failure. Research indicates that ferroptosis contributes to the progression of diabetic cardiomyopathy in diabetic models. For example, in db/db mouse cardiomyocytes, GPX4 protein expression decreased, whereas MDA and ACSL4 expression increased, and SLC7A11 expression decreased [93]. Ni et al. reported a notable reduction in FTH1 and GPX4 proteins, along with an elevated level of 4-HNE in the myocardial tissue of db/db mice, which could be ameliorated by Fer-1 [94]. Similarly, Wu et al. reported that diabetic cardiomyopathy mice induced by STZ, along with H9c2 cells induced by HGHF, exhibited increased iron content, elevated secretion of FACL4 protein, and decreased levels of GPX4 [95]. In addition, Chen et al. identified DEGs between the hearts of diabetic db/db mice and nondiabetic db/+ mice, revealing significant enrichment in iron ion binding, which suggests a potential role of ferroptosis in T2DM-related cardiac injury [96]. Luo et al. found that cardiac microvascular endothelial cells isolated from T2DM mice exhibited increased expression of pro-ferroptotic genes (Tfrc, Acsl4, Ptgs2), decreased expression of anti-ferroptotic genes (Gpx4, Slc7a11), and elevated lipid peroxidation (MDA, 4-HNE), indicating that hyperglycemia also induces ferroptosis in endothelial cells [97].

Recent studies have identified various genes and molecules as key contributors to iron-mediated cardiac injury in individuals with DM. Ablation of CD74 mitigates myocardial structural and functional damage in T2DM mice, with in vitro evidence indicating that CD74 ablation protects against T2DM-induced cardiac remodeling and systolic dysfunction via NLRP3/pyroptosis-mediated regulation of ferroptosis [96]. However, it should be noted that the extensive expression of this gene in immune cells may lead to compensatory changes in the systemic inflammatory network. Ni et al. demonstrated that both db/db mice and cardiomyocyte models treated with high glucose presented upregulated ZFAS1 expression and increased ferroptosis, whereas ZFAS1 inhibition reduced ferroptosis in the myocardium [94]. Tian et al. knocked out the mouse clock gene rev-erbα in the presence of HFD/STZ and reported that this exacerbates myocardial damage and ferroptosis in mice with T2DM [98]. Wang et al. found that NLRP3 is involved in the occurrence of myocardial ferroptosis in diabetic cardiomyopathy, and inhibiting NLRP3 both in vivo and in vitro can suppress diabetic-induced myocardial ferroptosis and trigger myocardial injury [99]. FGF21 binds to the heavy and light chains of ferritin, thereby reducing its excessive degradation via the proteasomal and lysosomal autophagic pathways in diabetic cardiomyopathy, causing diabetic cardiomyopathy [100]. The ferroptosis-related genes HSPB1 and MGST1, which are closely associated with immune cell infiltration, show that their downregulation significantly increases ferroptosis in diabetic myocardium and may serve as therapeutic targets [101]. The PACS2/CPT1A/DHODH signaling pathway may be involved in ferroptosis in diabetic cardiomyopathy by regulating mitochondrial function in cardiomyocytes [102]. The DNA–PK complex also serves as a key regulator of hyperglycemia-induced endothelial ferroptosis in T2DM cardiomyopathy, potentially representing a novel therapeutic strategy to alleviate T2DM microvascular dysfunction and cardiac dysfunction [97]. HMOX1 expression is upregulated in diabetic myocardial tissues, and knocking down HMOX1 ameliorates ferroptosis, thereby relieving diabetic cardiomyopathy by reducing cardiac fibrosis and improving cardiac function [103]. Additionally, previous research has shown that elevated blood sugar levels activate p53 [104, 105], which enhances cellular sensitivity to ferroptosis by suppressing SLC7A11 expression and reducing cystine uptake [106]. Comparable alterations were noted in the aortic endothelium of db/db mice [107]. Tang et al. found that irisin-mediated p53 reduction decreases ferroptosis and protects cardiomyocytes against high-glucose injury by increasing SIRT1, decreasing p53 K382 acetylation to promote p53 degradation, and consequently upregulating SLC7A11 and GPX4 [108].

Certain drugs enhance ferroptosis in diabetic cardiomyopathy by targeting the NRF2 pathway. For example, treatment with sulforaphane, an NRF2 agonist, significantly attenuated ferroptosis in HGHF-cultured H9c2 cells [98]. Research has indicated that 6-gingerol can mitigate ferroptosis and inflammation in DM models via the NRF2/HO-1 pathway [95]. Interestingly, Tian et al. reported that ferroptosis exacerbates diabetic myocardial ischemia‒reperfusion injury (IRI) both in vitro and in vivo. Alterations in Fe^2+^, SOD, MDA, and mRNA levels in cell supernatants, alongside changes in ferroptosis marker protein expression, support the positive impact of the NRF2/FPN1 pathway on iron metabolism and ferroptosis-related diabetic myocardial IRI [109]. Wang et al. reported that exogenous H_2_S modulates the NRF2/GPX4/GSH pathway by enhancing Syvn1-Keap1 interactions, which in turn mitigates ferroptosis and mitochondrial apoptosis linked to oxidative stress and antioxidant pathways in diabetic mouse hearts [110]. In addition to the above, isorhapontigenin mitigates diabetic cardiac microvascular injury by inhibiting mitochondria-associated ferroptosis via the PRDX2/MFN2/ACSL4 pathway [111]. Baicalin inhibits ferroptosis in diabetic cardiomyocytes via activation of the SENP1/SIRT3 signaling pathway, alleviating diabetic cardiomyopathy in both in vitro and in vivo models [112]. Dietary capsaicin suppresses ferroptosis by activating TRPV1 and the Nrf2/HMOX1 pathway, thereby alleviating cardiac injury after myocardial infarction in T2DM mice [113]. Retinoic acid improves diabetic vascular endothelial dysfunction by inhibiting PI3K/AKT/YAP-mediated ferroptosis [114]. Additionally, Schisandrol B alleviates diabetic cardiac injury by upregulating p53 to inhibit ferroptosis and improve lipid metabolism [115].

#### Ferroptosis in the kidney of diabetic models

Prolonged high blood sugar can damage the glomeruli and renal tubules, gradually leading to impaired kidney function, known as diabetic nephropathy. Studies have indicated that ferroptosis contributes to the development of diabetic nephropathy. Diabetic nephropathy models were established using STZ and db/db mice. Notable alterations in ferroptosis markers were observed, characterized by reduced GPX4 expression and elevated levels of SLC7A11, SLC3A2, MDA, and ACSL4 [116]. In vitro experiments demonstrated that ferroptosis inducers such as erastin or RSL3 induce renal tubular cell death, with the presence of iron and elevated ACSL4 levels increasing ferroptosis sensitivity [116]. Through single-cell transcriptomics, Tsai et al. reported that ferroptosis contributes to diabetic nephropathy progression and that ceruloplasmin centrally regulates ferroptosis in the proximal renal tubular cells of db/db mice expressing AQP4. This may lead to impaired cell repair and regulatory dysfunction of the renin‒angiotensin system in early diabetic nephropathy [117]. In addition, Wu et al. reported that both the ferroptosis inducer erastin and high glucose levels can trigger ferroptosis in mesangial cells [61].

NRF2 serves as a key therapeutic target for diabetic nephropathy via ferroptosis inhibition, exhibiting downregulated expression in diabetic models. Pharmacological agents such as fenofibrate, carnosine, umbelliferone, paricalcitol, leonurine, triptolide, DDO-1039, and Potentilla Discolor Bunge(whose main active components are quercetin, kaempferol, and β-sitosterol) can upregulate NRF2, thereby suppressing ferroptosis and decelerating the progression of diabetic nephropathy [118–126]. Additionally, swietenine mitigates diabetic nephropathy progression by activating the Akt/GSK-3β/Nrf2 signaling axis, which alleviates oxidative stress and restrains ferroptosis [127]. Moreover, knockdown of HMGB1 or S1R, as well as overexpression of Sirt6, alleviates diabetic nephropathy by regulating the NRF2 pathway, indicating the potential of NRF2 as a research target [61, 128, 129].

Research has also identified the HIF1-α/HO1 pathway as a critical target for ferroptosis in diabetic nephropathy. DM accelerated tubular iron accumulation and increased the levels of HIF1-α and HO1 both in vitro and in vivo. Conversely, the ferroptosis inhibitor Fer-1 and the sodium‒glucose cotransporter 2 (SGLT2) inhibitor dapagliflozin can suppress overactivation of the HIF1-α/HO1 axis [130, 131]. In addition, Shen Kang Pill may mitigate ferroptosis-induced kidney damage by inhibiting the HIF-1α/HO-1 signaling pathway [132].

In addition, other targets may improve ferroptosis in diabetic nephropathy. ARNTL is overexpressed in db/db mice, while the knockdown of ARNTL reduces oxidative stress in plasma and improves the classical morphological changes associated with renal ferroptosis [81]. In db/db mouse kidney tissue, SNHG1, ACSL4, and miR-16-5p expression levels are abnormal but can be normalized by Fer-1 treatment or SNHG1 knockdown in both db/db mice and high glucose-treated HK-2 cells [133]. HINT2 mitigated ROS-induced oxidative stress and ferroptosis associated with the MCU protein, as reported by Bai et al. HINT2 expression was downregulated in the kidney tissue of diabetic mice induced by HFD/STZ [82]. Additionally, Xiong et al. demonstrated that RAC1 overexpression notably enhanced ferroptosis in the kidneys of STZ-induced diabetic mice and high glucose–treated podocytes. Conversely, Rhein was found to inhibit ferroptosis and epithelial‒mesenchymal transition by modulating the RAC1/NOX1/beta-catenin axis, thus mitigating diabetic nephropathy [134]. The study by Liu et al. demonstrated that high glucose significantly promotes TGF-β1 secretion in HK-2 renal tubular cells, triggering ferroptosis via the TGF-β1/Smad3 signaling pathway, while sleeve gastrectomy alleviates the symptoms and progression of diabetic kidney disease by downregulating this pathway [135]. Chen et al. found that canopy FGF signaling regulator 2 (CNPY2) activates the PERK/ATF4/CHAC1 signaling pathway to promote ferroptosis, thereby leading to tubular injury in diabetic nephropathy [136]. Peng et al. discovered that KAT2A promotes diabetic nephropathy by upregulating H3K79succ and SAT2 to facilitate inflammation and ferroptosis [137]. Moreover, Yao et al. demonstrated that targeting NADPH oxidase-mediated ROS release and ferroptosis accumulation can improve septic kidney injury in LPS-stimulated HFD-induced diabetic mice and may also be a potential therapeutic target [138].

Canagliflozin and dapagliflozin are commonly used antidiabetic drugs that inhibit SGLT2 to reduce the reabsorption of glucose by the kidney. Research has also revealed that SGLT2 can independently inhibit ferroptosis in diabetic nephropathy. A study by Huang et al. initially explored the role of dapagliflozin in inhibiting ferroptosis, revealing that it reduces ubiquitination and degradation by binding to SLC40A1, independent of SGLT2 inhibition [139]. Coincidentally, Gan et al. demonstrated that canagliflozin inhibits ferroptosis in diabetic mouse kidneys via the FOXA1–CPT1A axis and reduces ferroptosis in HK-2 cells induced by high glucose levels [140]. Although the above studies have shown that SGLT2 inhibitors suppress ferroptosis through glucose-lowering independent mechanisms, clinical trials have only observed delayed progression of kidney disease, without confirming direct regulation of ferroptosis markers.

In addition, various other drugs, such as calycosin, the ACSL4 inhibitor rosiglitazone, dulaglutide, cordycepin, kidney tea (*Orthosiphon aristatus* (Blume) Miq.), and curcumin nanocrystals (a NRF2 agonist), as well as acupuncture, exert renoprotective effects in diabetic kidney disease by modulating ferroptosis [116, 141–146]. Vitamin D mitigates renal injury in prediabetic mice by activating the Klotho/p53 signaling pathway to suppress ferroptosis [147]. Liraglutide inhibits ferroptosis via the Fsp1-CoQ10-NAD(P)H pathway to alleviate renal fibrotic injury in db/db mice [148]. Hirsutine alleviates ferroptosis in podocytes of db/db mice with diabetic nephropathy by downregulating the p53/GPX4 signaling pathway [149]. The Qing-Re-Xiao-Zheng-(Yi-Qi) formula attenuates DKD progression by activating the AMPK signaling pathway, thereby inhibiting podocyte ferroptosis [150].

#### Ferroptosis in the liver of diabetic models

Ferroptosis contributes to liver pathology in DM. Altamura et al. demonstrated that increased liver iron levels in mice lead to oxidative stress, lipid peroxidation, and worsened insulin resistance, thereby exacerbating DM-related liver complications [151]. Iron deposition in the livers of STZ-induced diabetic mice was observed; this effect was mitigated by Fer-1 treatment, indicating the role of ferroptosis in DM-related liver pathology [152]. In the liver of db/db mice and HG-induced HepG2 cells, ferroptosis was observed, characterized by iron accumulation accompanied by decreased SLC7A11 expression, increased activities of SOD, GSH-PX, and GSH, as well as elevated levels of MDA, 4-HNE, NOX4, and ROS [153]. Mechanistically, Meng et al. found that ZHX2 levels were lower and YTHDF2 levels were higher in the liver of HFD/STZ-induced diabetic mice and HG-induced Huh7 cells. They demonstrated that ZHX2 knockdown increased ferroptosis in Huh7 cells by activating YTHDF2-induced degradation of GPX4 and SLC7A11, suggesting a potential targeted therapeutic pathway [154]. Notably, liraglutide can mitigate ferroptosis and enhance the previously mentioned alterations by modulating the NRF2/HO-1/GPX4 signaling pathway [153].

#### Ferroptosis in the brain of diabetic models

DM-induced cognitive dysfunction is a neurological complication associated with DM. In vivo and in vitro studies suggest that ferroptosis may contribute to DM-related cognitive dysfunction, causing neuronal damage in diabetic animals. This study revealed that mitochondrial and neuronal damage, along with iron overload and ferroptosis, occur in the hippocampus of STZ-induced type 1 diabetic rats, indicating that SLC40A1-mediated ferroptosis is linked to cognitive impairment in DM [155]. Ferroptosis has been identified in the hippocampus of the HFD/STZ-induced T2DM model, as reported by Xie et al. Research indicates that ferroptosis predominantly occurs in hippocampal neurons rather than in microglia or astrocytes and is characterized by increased transferrin receptors and reduced levels of ferritin, GPX4, and SLC7A11 in these neurons [156–158]. These findings suggest that activating AMPK decreased hippocampal ferroptosis and enhanced cognitive function in diabetic mice [157]. Tang et al. identified that caveolin-1, a membrane protein that plays a role in cell signaling and material transport, was significantly downregulated in the hippocampus of diabetic mice, and its overexpression can mitigate the cognitive dysfunction linked to DM by modulating neuronal ferroptosis and maintaining mitochondrial homeostasis [60]. Feng et al. found that the ferroptosis-related gene ALOX15 was significantly upregulated in neurons of STZ-induced T1DM mice and HG-treated SH-SY5Y cells, promoting the development of diabetic peripheral neuropathic pain by enhancing neuronal ferroptosis [159].

Astrocytes are essential for brain energy metabolism and maintaining antioxidant levels. In contrast to the findings of Xie et al., Wang et al. demonstrated that high glucose induces ferroptosis in astrocytes by altering iron metabolism and suppressing the SLC7A11/GPX4 pathway in db/db mice [160]. The discrepancy between the two studies may be attributed to the different molecular mechanisms related to models, as the STZ-induced diabetic model employed by Xie et al. leads to pancreatic islet β cell damage, whereas the db/db mouse model is deficient in leptin receptors. Furthermore, in vitro studies have demonstrated that high-glucose conditions dose-dependently suppress PPARα activity in astrocytes, increasing their susceptibility to ferroptosis, and gemfibrozil, a PPARα agonist, inhibits ferroptosis in db/db mice and astrocytes [160]. Additionally, Zhao et al. observed ferroptosis in microglia under high-glucose conditions and confirmed that TREM1 exacerbates iron accumulation in T2DM-related microglia via the PERK pathway of ERS, leading to antioxidant inactivation, lipid peroxidation, and significant ROS increase that induces microglial ferroptosis [161].

Additionally, several other drugs exert anti-ferroptosis effects and thereby improve cognitive dysfunction associated with DM. Liraglutide mitigates oxidative stress, lipid peroxidation, and iron overload in diabetic patients with cognitive disorders, inhibiting ferroptosis and reducing damage to hippocampal neurons and synaptic plasticity, ultimately restoring cognitive function [156]. Artemisinin inhibits neuronal ferroptosis in the hippocampus by activating NRF2, effectively improving neuropathological changes and learning-memory decline in T2DM mice [162]. Dendrobine ameliorates cognitive dysfunction in diabetic encephalopathy by inhibiting ferroptosis through activation of the NRF2/GPX4 pathway in db/db mice and AGE-induced HT22 cell models [163]. The total alkaloids of Rhizoma Corydalis effectively reduce NLRP3-induced ferroptosis, alleviate pathological damage in the hippocampus of diabetic rats, and thereby improve cognitive function [164]. Salidroside improves diabetic cognitive impairment in db/db mice by directly binding to PPARγ to promote its expression, thereby inhibiting oxidative stress and reducing neuronal ferroptosis [165]. Resveratrol protects against cognitive dysfunction and insulin resistance in T2DM by reducing extracellular vesicle- and miR-9-3p cargo-induced oxidative stress, inflammation, and ferroptosis in the ER and hippocampus [166].

#### Ferroptosis in the retina of diabetic models

Both in vivo and in vitro models of diabetic retinopathy exhibit lipid peroxidation, oxidative stress, and ferroptosis. Fan et al. observed ferroptosis in the retinas of STZ-induced diabetic model mice and HG-treated ARPE-19 cells, and found that inhibiting FABP4 reduces lipid peroxidation and oxidative stress in diabetic retinopathy by regulating PPARγ-mediated ferroptosis [167]. Wang et al. further found that ferroptosis occurred in both retinal and occipital lobe neurons of STZ-induced diabetic rats, leading to diabetic-induced visual pathway neuronal injury through iron accumulation and GPX4 inactivation [162]. Zhang et al. determined that ferroptosis is a key mechanism underlying blood-retinal barrier damage in diabetic retinopathy, demonstrating that Flotillin-1 activates the Nrf2 pathway by enhancing its expression and promoting its nuclear translocation, thereby stimulating the SLC7A11/GPX4 pathway to inhibit lipid peroxidation and ferroptosis [168]. Additionally, Liao et al. discovered that the m⁶A demethylase ALKBH5 delays the progression of diabetic retinopathy by inhibiting ferroptosis via the m⁶A–YTHDF1–ACSL4 axis, suggesting its potential as a therapeutic target [169].

Some studies have shown that several drugs can inhibit ferroptosis and improve diabetic retinopathy. Corilagin alleviates ferroptosis in diabetic retinopathy by activating the Nrf2 signaling pathway [170]. Resveratrol alleviates reactive oxygen species and inflammation in diabetic retinopathy via the SIRT1/HMGB1 pathway, which regulates ferroptosis [171]. Carnosic acid prevents retinal ferroptosis and alleviates diabetic retinopathy by activating the SIRT1/p53/SLC7A11 pathway in both in vivo and in vitro models [172]. Mesenchymal stem cell-derived small extracellular vesicles (MSC-sEVs) exhibit therapeutic effects by maintaining vascular endothelial function, and sEVs carrying miR-125b-5p prevent endothelial cell ferroptosis by inhibiting p53 and thereby protect the blood-retinal barrier [173]. Additionally, Chen et al. concluded that fenofibrate can inhibit ferroptosis to improve diabetic retinopathy, but did not measure iron-related indices closely associated with ferroptosis, such as iron levels, GPX4, and SLC7A11, indicating that the results require further confirmation [174].

#### Ferroptosis in tendons and bone of diabetic models

The diabetic microenvironment significantly contributes to the development of tendon pathology. However, the mechanisms underlying tendon pathology in patients with DM remain unclear. Notably, HGHF-treated tenocytes distinctly exhibited a ferroptotic phenotype characterized by lipid peroxide buildup, changes in mitochondrial structure, decreased mitochondrial membrane potential, iron accumulation, and upregulation of ferroptosis-associated genes [175]. In animal studies, db/db mice exhibited severe tendon injury along with elevated ACSL4 and TfR1 expression, potentially activated by YAP [175].

T2DM is frequently associated with osteoporosis, a condition that accelerates bone loss and elevates fracture risk. Xu et al. demonstrated that ferroptosis occurs in HGHF-induced bone mesenchymal stem cells and bones of HGHF/STZ-induced diabetic model mice, characterized by elevated ROS and ACSL4 levels alongside GPX4 expression. Xu et al. further reported that poliumoside counteracts HGHF-induced bone degradation and ferroptosis by activating the NRF2/GPX4 signaling pathway, suggesting its potential as a novel therapeutic approach for T2DM-associated osteoporosis [176]. Dong et al. demonstrated that ferroptosis, accompanied by decreased FNDC5 expression, occurs in both STZ-induced type 1 diabetic mouse bone tissue and high glucose-exposed MC3T3-E1 cells, with irisin administration or FNDC5 overexpression ameliorating type 1 diabetic bone loss via inhibition of endoplasmic reticulum stress-mediated ferroptosis [177].

#### Ferroptosis in testes and penis of diabetic models

Erectile dysfunction and germ cell dysfunction are recognized complications of DM, and emerging evidence highlights that ferroptosis plays a critical role in mediating DM-induced testicular and penile injury. Cao et al. found that ferroptosis occurred in testicular tissue of STZ-induced diabetic mice and in high glucose-treated GC-2 testicular glial cells, both of which showed iron accumulation, increased MDA levels, decreased GSH levels, and down-regulated GPX4, FTL, and SLC7A1 [63]. Xiao et al. also observed similar phenomena in the testes of STZ-induced diabetic mice and HG-treated GC-1spg cells, and found that BRD7 inhibits *Clusterin* expression by regulating hypermethylation of the *Clusterin* promoter in an EZH2-dependent manner, thereby suppressing AMPK signaling to promote ferroptosis and induce DM-related testicular injury [178]. In addition, Fu et al. discovered that Guhan Yangsheng Jing, a traditional Chinese patent medicine primarily composed of *Polygonatum sibiricum*, *Epimedium*, and *Lycium barbarum*, ameliorates reproductive damage in diabetic male rats by upregulating the Nrf2/HO-1 pathway to alleviate oxidative stress and ferroptosis [179].

Accumulating evidence indicates that ferroptosis occurs in the penile tissues of diabetic models. Ferroptosis was observed in penile tissues of diabetic rats and HG-treated cavernous endothelial cells, and isorhamnetin improved erectile function in diabetic rats by inhibiting ferroptosis and oxidative stress [180]. Yang et al. focused on the proportion of corpus cavernosum cell death in STZ-induced T1DM rats and found ferroptosis in the corpus cavernosum tissue, endothelial cells, and smooth muscle cells under high-glucose conditions, while icariin improved erectile function in T1DM rats by suppressing oxidative stress and subsequently inhibiting ferroptosis, apoptosis, and pyroptosis in these cells [181]. Similarly, hesperidin ameliorates diabetic erectile dysfunction in rats by activating the Nrf2/HO-1/GPX4 pathway in endothelial and smooth muscle cells to inhibit ferroptosis [182].

#### Ferroptosis in the skin of diabetic models

Diabetic patients often experience impaired skin wound healing due to hyperglycemia-induced mechanisms such as vascular endothelial dysfunction, reduced immune function, impaired keratinocyte activity, and oxidative stress. Studies have revealed the presence of ferroptosis at wound sites in diabetic rats, and histone lysine crotonylation accelerates ACSL4-mediated ferroptosis in keratinocytes via autophagy regulation, thereby influencing diabetic wound healing [183]. Jiang et al. observed ferroptosis in AGEs-induced HaCaT cells and found that berberine alleviates AGEs-induced ferroptosis by activating NRF2 in the skin of diabetic mice [184]. Additional studies have shown that orientin, dulaglutide, and dracorhodin inhibit ferroptosis by activating the Nrf2 pathway, thereby promoting diabetic wound healing [185–187].

Furthermore, alterations in lipid peroxidation biomarkers (lipid ROS, GPX4, SLC7A11, ALOX5, and ASLC4) were noted in both the STZ-induced diabetic limb ischemia mouse model and human microvascular endothelial cells under diabetic limb ischemia conditions, indicating a potential role of ferroptosis in diabetic limb ischemia. Further research revealed that the overexpression of AURKA mitigates diabetic limb ischemia by enhancing ferroptosis and inhibiting oxidative stress and lipid peroxidation both in vitro and in vivo [188].

#### Ferroptosis in the metabolism of the intestinal flora in diabetes

In addition to its role in the aforementioned tissues in the diabetic model, ferroptosis is also associated with intestinal flora metabolism in DM. A study examining the link between the gut microbiota and iron metabolism revealed that genera such as *Rikenella*, *Alistipes*, and *Odoribacter* were inversely associated with serum iron, blood glucose, weight, and transferrin levels. Conversely, genera such as *Lachnospiraceae UCG-001*, *Ruminococcus-1*, *Candidatus Arthromitus*, *Enterobacter*, and *Lactobacillus* exhibited positive correlations with these parameters [189]. Salidroside potentially lowers blood glucose in diabetic mice by altering the gut microbiota and modulating iron metabolism, offering protection against diabetic cardiomyopathy [189]. Another study revealed that astragaloside IV (AS-IV) treatment can significantly improve intestinal microflora dysregulation in db/db mice, marked by increased abundances of *Akkermansia*, *Ligilactobacillus*, and *Lactobacillus*. This study revealed that AS-IV notably decreased ferroptosis in kidney and colon tissues, implying that modifications in the gut microbiome and ferroptosis regulation might be mechanisms through which AS-IV enhances DM management via the enterorenal axis [190].

**Fig. 2:**
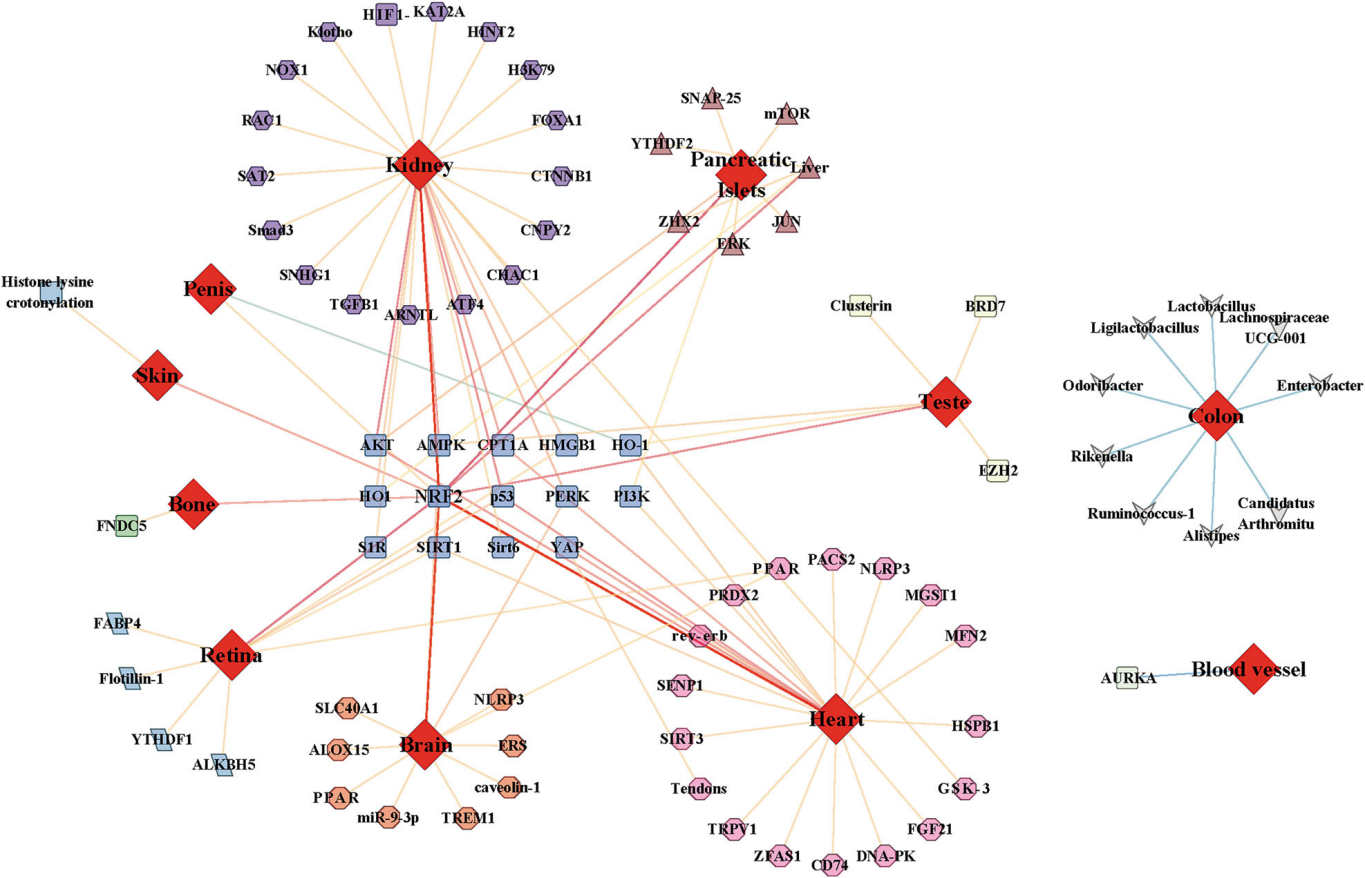
Targets regulated by high-glucose environment mediating ferroptosis in different tissues of diabetes.

**Fig. 3:**
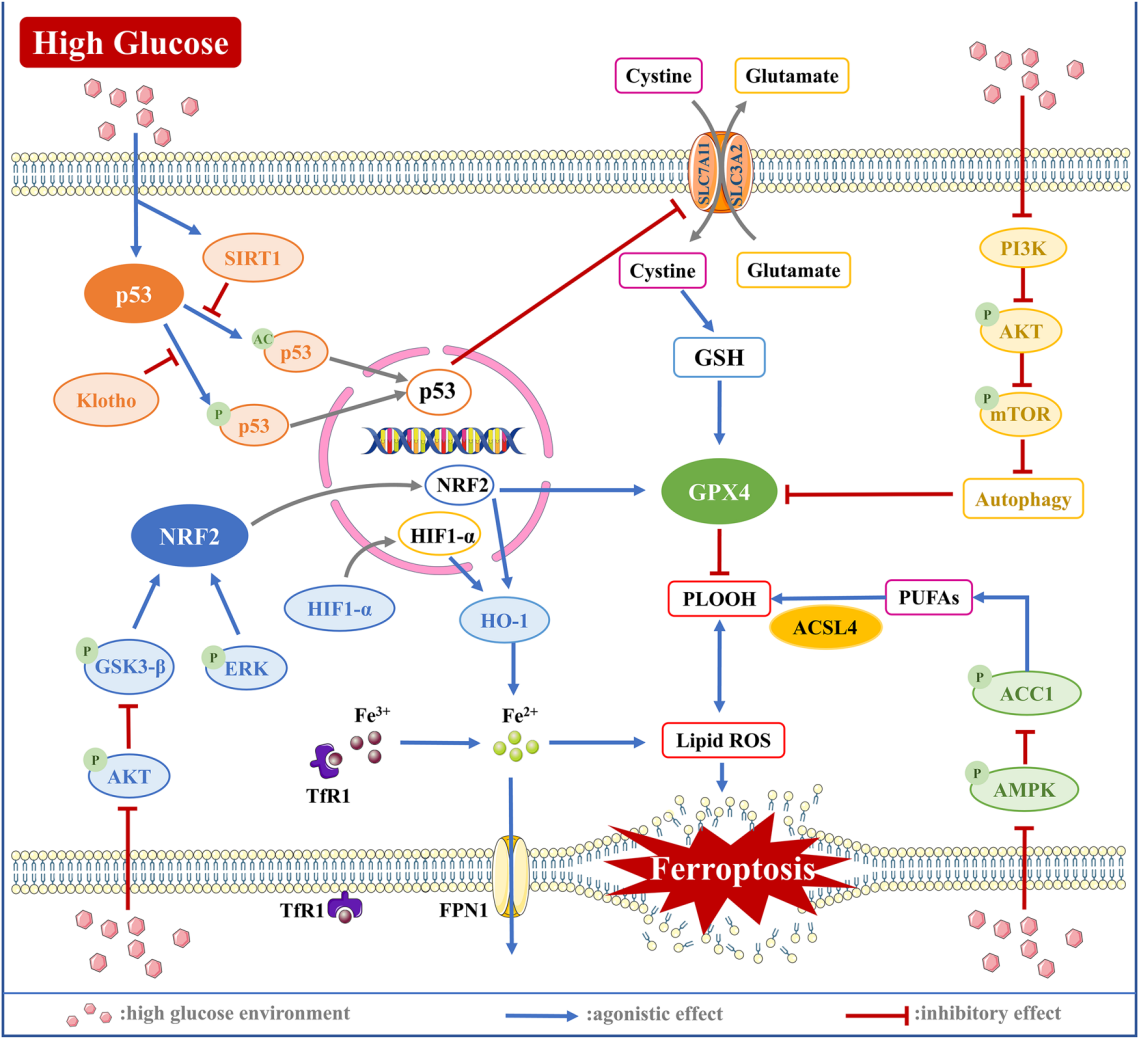
The classic molecular mechanism of ferroptosis in diabetic tissue cells under high-glucose conditions. The most studied target mediating ferroptosis in diabetes is NRF2, which exhibits decreased activity in cells. This occurs because high glucose suppresses the AKT/GSK3-β signaling pathway, further downregulating NRF2 activity. Consequently, the transcriptional expression of GPX4 and other antioxidant genes is inhibited, promoting ferroptosis. High glucose also activates p53, which enhances cellular sensitivity to ferroptosis by suppressing SLC7A11 expression, thereby reducing cystine uptake. Additionally, high glucose inhibits the PI3K/AKT/mTOR signaling pathway, inducing autophagy that suppresses GPX4 activity and increases intracellular iron content, further facilitating ferroptosis. Moreover, inhibition of the AMPK/ACC1 signaling pathway by high glucose promotes the production of PUFAs, accelerating lipid peroxidation and inducing ferroptosis.

Unlike prior studies emphasizing single-organ effects, our analysis reveals that ferroptosis drives a self-reinforcing cycle: Hyperglycemia in DM triggers systemic iron overload, and the accumulation of iron in peripheral organs, combined with oxidative stress imbalance, causes organ damage (including pancreatic β-cells), further exacerbating the progression of DM and forming a pathological feedback loop. By visualizing the molecular mechanisms involved in the previous text (Fig. 2), we found that almost all complications share a common mechanism (NRF2) for regulating ferroptosis, but due to the inherent characteristics of the tissues, they also have relatively unique regulatory methods. Furthermore, PI3K, wnt, and AMPK signaling may be the pathways with the highest association with other pathways. We summarize the key molecular mechanisms that drive the pathogenesis of diabetes and its associated complications in Fig. 3.

Based on the current evidence (Table 1), the clinical translation of ferroptosis-targeting therapeutics for DM and its complications remains a substantial challenge. As a shared therapeutic target for DM and its complications, NRF2 activation holds promise as a novel therapeutic strategy. Although dapagliflozin and canagliflozin demonstrate cardiorenal protective effects in clinical settings, systemic NRF2 activation may disrupt physiological redox homeostasis. Furthermore, gain-of-function mutations in KEAP1 or NFE2L2 polymorphisms could compromise drug responsiveness. The ferroptosis inhibitor Fer-1 reverses ferroptosis in diabetic cells and animal models, but its clinical translation is hindered by challenges such as poor pharmacokinetics and potential hepatotoxicity; similarly, the ACSL4 inhibitor rosiglitazone is restricted in clinical application due to risks of hepatotoxicity. Additionally, marketed antidiabetic drugs like metformin, canagliflozin, dapagliflozin, and liraglutide may exert part of their effects via ferroptosis inhibition but act through additional mechanisms. Moreover, other agents have only undergone preclinical trials, and notably, no clinical studies have yet demonstrated that inhibiting ferroptosis can improve DM.

## Conclusion and prospects

**Fig. 4:**
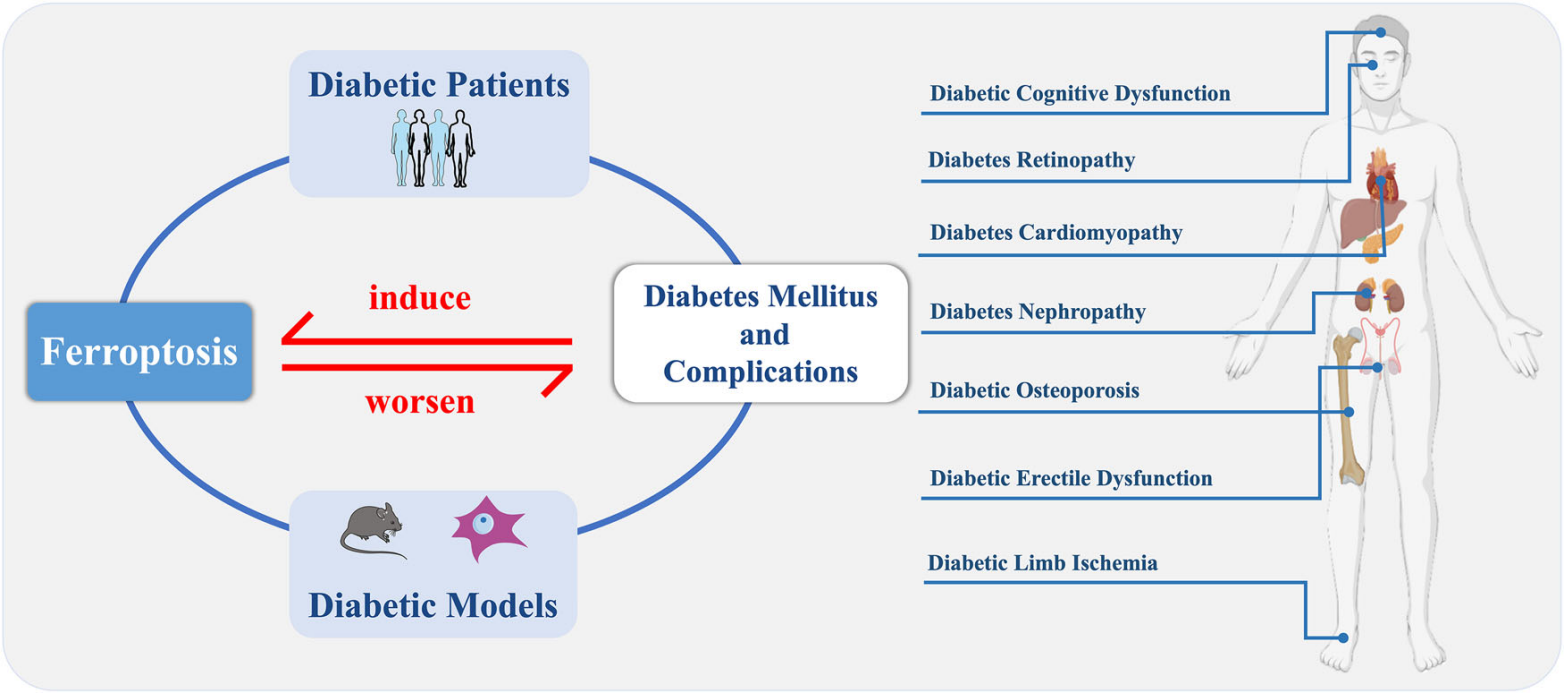
Through the correlation analysis between iron metabolism and diabetic patients, as well as the research on ferroptosis in diabetes-related models, it was found that the diabetic environment can induce the occurrence of ferroptosis, and ferroptosis can also exacerbate the development of diabetes and its complications.

This review establishes ferroptosis as a convergent pathological mechanism driving diabetic complications across multiple organs and as a pivotal nexus linking “iron dyshomeostasis-oxidative stress-multiorgan injury”(Fig. 4). Organ lipid peroxidation and mitochondrial damage mediated by oxidative stress constitute shared pathological processes underlying both ferroptosis and DM-associated organ injuries. Notably, the inherent susceptibility of pancreatic β-cells to ferroptosis provides a mechanistic basis for DM progression, while hyperglycemia reciprocally promotes systemic iron accumulation, thereby establishing a self-reinforcing cycle. These findings reveal ferroptosis-mediated pathways connecting localized tissue damage to systemic diabetic pathogenesis. Mechanistically, while NRF2-regulated ferroptosis emerges as a common denominator across complications, distinct molecular features characterize its organ-specific manifestations. This review validates various ferroptosis inhibition strategies (including NRF2 activation) through drug repurposing and novel agents, all of which have yielded suboptimal outcomes. To advance therapeutic translation, future research should prioritize: (i) Gender-stratified validation of clinical biomarkers for early detection; (ii) Development of highly effective and safe NRF2 activator or tissue-selective ferroptosis modulators; (iii) Elucidation of crosstalk mechanisms with metabolic pathways (e.g., PI3K/AKT, insulin signaling). Collectively, this synthesis proposes a strategic framework for ferroptosis-targeted interventions that extends beyond conventional glycemic control paradigms in DM management.
